# Estimating heritability of drug-induced liver injury from common variants and implications for future study designs

**DOI:** 10.1038/srep05762

**Published:** 2014-07-21

**Authors:** Casey Lynnette Overby, George Hripcsak, Yufeng Shen

**Affiliations:** 1 Department of Biomedical Informatics, Columbia University, New York, NY; 2 Department of Systems Biology, Columbia University, New York, NY; 3 JP Sulzberger Columbia Genome Center, Columbia University, New York, NY; 4 Current address: Program in Personalized & Genomic Medicine and Department of Medicine, University of Maryland, Baltimore, MD.

## Abstract

Recent genome-wide association studies identified certain human leukocyote antigen (HLA) alleles as the major risk factors of drug-induced liver injuries (DILI). While these alleles often cause large relative risk, their predictive values are quite low due to low prevalence of idiosyncratic DILI. Finding additional risk factors is important for precision medicine. However, optimal design of further genetic studies is hindered by uncertain overall heritability of DILI. This is a common problem for low-prevalence pharmacological traits, since it is difficult to obtain clinical outcome data in families. Here we estimated the heritability (*h*^*2*^) of DILI from case-control genome-wide single nucleotide polymorphism data using a method based on random effect models. We estimated the proportion of *h*^*2*^ captured by common SNPs for DILI to be between 0.3 and 0.5. For co-amoxiclav induced DILI, chromosome 6 explained part of the heritability, indicating additional contributions from common variants yet to be found. We performed simulations to assess the robustness of the *h*^*2*^ estimate with limited sample size under low prevelance, a condition typical to studies on idiosyncratic pharmacological traits. Our findings suggest that common variants outside of HLA contribute to DILI susceptability; therefore, it is valuable to conduct further GWAS with expanded case collection.

Given the relatively small sample sizes obtainable in pharmacogenetic studies^1^, consortia are forming with the goal of improving power to detect associations by pooling data generated from multiple studies. One effort relevant to this work is the International Serious Adverse Event Consortium (iSAEC). Phase 1 of the project has resulted in one of the largest DILI research collections in the world and initial studies have successfully identified genetic variants associated with DILI^2^.

While there are some successes identifying major risk factors of DILI (e.g., HLA-B*5701 as a major risk factor of liver injury due to flucloxacillin), no SNPs were identified in DILI across all drugs, nor have any been validated when flucloxacillin and coamoxiclav are excluded in this collection. This is likely due to limited power of detecting modest SNP effects with the relatively small cohort size. Understanding the heritability of these conditions is one step toward understanding genetic architecture of pharmacogenomic risk, and may provide valuable information for designing future studies.

Genome-wide association studies (GWAS) usually assume that common diseases are attributable to allelic variants present in more than 1–5% of the population^3^. However, most variants identified so far in GWAS analyses explain relatively small increases in risk. Also, in complex conditions where heritability has been estimated through twin and family studies, GWAS have failed to produce findings to support estimations. For example, the heritability of height is estimated to be 80%, however only 4% can be explained by variants identified in GWAS studies^4^. This discrepancy has been described as the missing heritability problem^5^. Proposed explanations for this issue are that rare variants with larger effects, gene-gene interactions, and/or gene-environment interactions are poorly detected by GWAS approaches^6^. Another explanation for the missing heritability is that common variants of smaller effect have not yet been identified. Peter Visscher's group has developed the Genome-wide Complex Trait Analysis (GCTA) method to measure the effect of common variants using a mixed model with genome-wide SNP data^7^. Applying this approach has provided further evidence that a substantial proportion of heritability is captured by common SNPs in complex traits including height and body mass index (BMI).

This approach is particularly attractive to apply in the context of pharmacogenomics studies, where ascertainment of family data is difficult or even impossible. Given the implicit assumption that adverse drug reactions (ADRs) are subject to substantial genetic control in pharmacogenomics studies, it is important to present evidence of the size of the genetic component (or heritability) for the trait under investigation.

Heritability estimates for complex traits are typically determined through twin studies. However, these approaches have limited utility in the context of susceptibility to ADRs^8^. Limitations are primarily due to difficulties recruiting and obtaining clinical outcome data in twins. This limitation might be overcome by applying the GCTA approach that estimates the effect of common variants from genome-wide SNP data. In this study we assessed the performance of this approach with DILI GWAS datasets.

## Results

### Proportion of heritability captured by genome-wide common variants for drug-induced liver injury

We estimated the proportion of *h*^*2*^ captured by all genome-wide common SNPs, chromosome 6 genome-wide SNPs and genome-wide SNPs without chromosome 6 for DILI. Results for liver injuries induced by co-amoxiclav and flucloxacillin are summarized in Table 1. We observe that for flucloxacillin induced DILI patients, chromosome 6 explained almost all of the heritability (*h*^*2*^_all_ = 0.48 ± 0.313, *h*^*2*^_chr6_ = 0.478 ± 0.076, *h*^*2*^_NOchr6_ = 0.18 ± 0.40). Whereas with co-amoxiclav induced DILI patients, chromosome 6 explained part of the heritability (*h*^*2*^_all_ = 0.40 ± 0.16, *h*^*2*^_chr6_ = 0.17 ± 0.041, *h*^*2*^_NOchr6_ = 0.378 ± 0.17), indicating contributions from additional common variants are yet to be found. Estimates for *h*^*2*^ may be underestimated due to GCTA algorithm constraints so that SNP-heritability on the observed scale could not be greater than 1. Even so, this finding suggests that continuous collection of DILI cases is valuable for the potential of discovering additional associations with common genetic variants.

### GCTA algorithm estimation of heritability for moderate sample sizes

The heritability of T1D is estimated from pedigree studies to be 0.9^9^,^10^. To date, the proportion of phenotypic variance explained by validated SNPs and genome-wide significant variants (including pre-GWAS loci with large effects) is 0.6^9^,^11^. In addition, the proportion of phenotypic variance explained when all GWAS SNPs are considered simultaneously is 0.3^9^,^12^. With the GCTA algorithm, we estimated the proportion of phenotypic variance explained by all genome-wide SNPs, chromosome 6 genome-wide SNPs, and genome-wide SNPs without chromosome 6. Results of data for all WTCCC data are summarized in Table 2. Results of data for 75 and 200 cases are summarized in Table 3. Our simulations to assess of the impact of sample sizes on heritability estimates indicated relatively stable estimates with moderate sample sizes. We estimate the proportion of *h*^*2*^ captured by common SNPs for T1D to be, on average, 0.51 ± 0.23 with a sample size of 75 and 0.46 ± 0.15 with a sample size of 200. These estimates are similar to estimations made with the entire dataset of 1963 cases and 2938 controls (h^2^_all_ = 0.44 ± 0.037). Moreover, estimates calculated from sample sizes of 75 and 200 cases gave similar results, with both indicating that chromosome 6 explains part of the heritability (the *h*^*2*^*_chr6_* estimate is 0.19 ± 0.15 with 75 cases and 0.12 ± 0.058 with 200 cases). For a sample size of 75, however, the standard deviation for our *h*^*2*^*_chr6_* estimate is close to the mean. This indicates that calculations are not stable with smaller sample sizes, underscoring the uncertainty about flucloxacillin-induced DILI. Estimates for *h*^*2*^*_chr6_* calculated using this particular T1D dataset may also be underestimated due to inadequate SNP density in the MHC region in this particular data set.

### Analysis of measurement errors in the drug-induced liver injury dataset

Lastly, we observe that the estimated *h*^*2*^ is 0 for both flucloxacillin induced and co-amoxiclav induced DILI controls coded as cases. Results are summarized in Table 4. We conclude that there were no substantial measurement errors in our datasets.

## Discussion

While further investigation is required to confirm the robustness of the GCTA algorithm for low prevalence traits such as DILI, this work highlights the potential value of its application. Here we were able to apply the algorithm to investigate the contribution of common variants on the chromosome level. Such investigations can provide insight into disease mechanism and into inter-individual variation. Moreover, for ADRs in particular, we are able to provide previously unobtainable estimates of heritability. Such estimates will help optimize designs of future studies for identifying additional genetic contributions to these conditions.

Findings suggest that collecting and genotyping more co-amoxiclav induced DILI cases is valuable for discovering additional associations. While further investigation is required to confirm the robustness of the GCTA algorithm for low prevalence traits like DILI, this work highlights the potential value of its application. Particularly for pharmacological traits, we can provide previously unobtainable estimates of heritability. Such estimates will help optimize designs for future studies.

## Methods

### Study population

DILI datasets were from case-control studies of individuals taking flucloxacillin (77 cases and 288 population controls)^2^ and individuals taking co-amoxiclav (201 cases and 532 population controls)^13^. The genotyping data were generated using the Illumina 1M-duo described previously^2^,^13^.

### Genome-wide complex trait analysis algorithm

The GCTA algorithm measures the effect of common variants with genome-wide SNP data using a mixed model approach^7^. The algorithm involves first estimating the genetic relationship matrix (GRM) of all individuals, then fitting the GRM in a mixed linear model (MLM) for binary traits to estimate the proportion of variance explained by all the autosomal SNPs. We used GCTA algorithm extended for case-control designs^14^ to estimate *h*^*2*^ captured by common SNPs for DILI. We also assessed the robustness of the GCTA algorithm with datasets of moderate sample sizes and low prevalence.

### Estimating proportion of heritability captured by common variants

We used the GCTA algorithm to estimate *h*^*2*^ from iSAEC DILI genome-wide data (all chromosomes), chromosome 6 data, and genome-wide data without chromosome 6. We also estimated *h*^*2*^ for all individuals (co-amoxiclav or flucloxacillin induced DILI), for individuals with co-amoxiclav induced DILI, and for individuals with flucloxacillin induced DILI. For individuals with co-amoxiclav induced DILI we evaluated northwest Europeans only and southern Europeans both together and separately. All analyses were performed separately. For each population and dataset, unless otherwise noted, we calculated *h*^*2*^ adjusting for prediction errors due to global structure and local structure. We used principal components analysis (PCA) to adjust for prediction errors due to global structure. Two principal components were included as regression covariates in the mixed model. We also used an optional functionality of the GCTA tool to adjust for prediction errors due to imperfect linkage disequilibrium. All analyses were performed for DILI prevalence estimated to be 0.0001^2^,^15^ and 0.0005. We reported main results using a prevalence of 0.0005 based on recent work (the estimated rate is 0.00043 in Iceland population^16^) and a general trend of underreporting of DILI incidences^17^.

### Assessing the robustness of the GCTA algorithm with moderate sample sizes

To evaluate the robustness of the GCTA algorithm with datasets of moderate sample sizes, we conducted a positive control and negative control experiment. We performed simulations for our positive control experiment. Simulations involved estimating *h*^*2*^ for subsets of cases and controls from a Type I Diabetes (T1D) dataset. We choose to use a T1D because the histocompatibility complex (MHC) region on chromosome 6 has major genetic contribution to risks of both DILI^2^ and T1D^11^,^18^. Specifically, we used the Welcome Trust Case Control Consortium (WTCCC) T1D dataset (1963 cases and 2938 controls)^18^. Genotyping was conducted for the WTCCC study using an Illumina 550K chip. We estimated *h*^*2*^ from genome-wide data (all chromosomes), chromosome 6 data, and genome-wide data without chromosome 6 for cases and controls with a 1:3 ratio, where the number of cases were 75 and 200 to reflect sample sizes similar to iSAEC DILI sample sizes. Estimates for *h*^*2*^ were averaged over twenty random selections of cases and controls for these sample sizes. The prevalence of T1D is estimated to be 0.008 19. We report the mean and standard deviation of the twenty *h*^*2*^ estimates. We also report *h*^*2*^ estimates and standard errors with the full WTCCC T1D dataset and the GCTA parameter for prevalence set at 0.0005 and 0.008. Given the WTCCC data were collected from a homogenous population, however, we do not adjust for prediction errors due to global structure.

For a negative control experiment we tested for measurement errors by estimating *h*^*2*^ for DILI controls coded as cases with WTCCC controls. This analysis was performed with northwestern European controls from the flucloxacillinin induced and co-amoxiclav induced DILI datasets. The prevalence of DILI was set to be 0.0005.

## Author Contributions

C.L.O. and Y.S. designed the study, analyzed and interpreted the data, and wrote the manuscript. C.L.O. performed the experiments. G.H. interpreted the data and critically revised the manuscript for important content. All authors approved the final version of the manuscript.

## Figures and Tables

**Table 1:** Heritability estimates from iSAEC genome-wide data for co-amoxiclav and flucloxacilin induced drug-induced liver injury

Drug (population)	Prevalence	No. cases	No. controls	Estimated *h^2^_all_* (SE)	Estimated *h^2^_chr6_* (SE)	Estimated *h^2^_NOchr6_* (SE)
co-amoxiclav	0.0005	201	532	0.40 (0.16)	0.17 (0.041)	0.38 (0.17)
flucloxacillin	0.0005	77	288	0.48 (0.32)	0.48 (0.076)	0.18 (0.40)
co-amoxiclav	0.0001	201	532	0.26 (0.10)	0.11 (0.027)	0.25 (0.11)
flucloxacillin	0.0001	77	288	0.31 (0.21)	0.31 (0.05)	0.12 (0.26)

**Table 2:** Heritability estimates from WTCCC genome-wide data for T1D

T1D Prevalence	No. cases	No. controls	Estimated *h^2^_all_* (SE)	Estimated *h^2^_chr6_* (SE)	Estimated *h^2^_NOchr6_* (SE)
0.0005	1963	2938	0.26 (0.022)	0.068 (0.008)	0.22 (0.022)
0.008	1963	2938	0.434 (0.037)	0.11 (0.014)	0.37 (0.037)

**Table 3:** Heritability estimation from WTCCC genome-wide data for T1D with limited number of samples

T1D Prevalence	No. Cases	No. Controls	Estimated *h^2^_all_* (SD)	Estimated *h^2^_chr6_* (SD)	Estimated *h^2^_NOchr6_* (SD)
0.008	75	225	0.51 (0.23)	0.19 (0.15)	0.28 (0.31)
0.008	200	600	0.46 (0.18)	0.12 (0.058)	0.35 (0.16)

**Table 4:** Heritability estimation to assess measurement errors

DILI indicated drug (population)	No. cases	No. controls	Estimated *h^2^_all_* (SE)
flucloxacillin	288 (controls coded as cases)	864 (WTCCC controls)	0.0 (0.11)
co-amoxiclav	306 (controls coded as cases)	918 (WTCCC controls)	0.0 (0.11)
